# Seeing the value of experiential knowledge through COVID-19

**DOI:** 10.1007/s40656-021-00438-y

**Published:** 2021-06-29

**Authors:** Sarah Atkinson, Hannah Bradby, Mariacarla Gadebusch Bondio, Anna Hallberg, Jane Macnaugthon, Ylva Söderfeldt

**Affiliations:** 1 Department of Geography/Institute for Medical Humanities, Durham University, Durham, UK; 2 Department of Sociology, Uppsala University, Uppsala, Sweden; 3 Institute for Medical Humanities, University Hospital Bonn, Bonn, Germany; 4 Department of Public Health and Caring Sciences, Uppsala University, Uppsala, Sweden; 5 Institute for Medical Humanities, Durham University, Durham, UK; 6 Department of History of Science and Ideas/Centre for Medical Humanities, Uppsala University, Uppsala, Sweden

**Keywords:** Experiential knowledge, Epistemology, Social inequality

## Abstract

Seeing the entwinement of social and epistemic challenges through COVID, we discuss the perils of simplistic appeals to ‘follow the science’. A hardened scientism risks excarbating social conflict and fueling conspiracy beliefs. Instead, we see an opportunity to devise more inclusive medical knowledge practices through endorsing experiential knowledge alongside traditional evidence types.

Instead of hardening scientism—that is an unreflective trust in scientific methods and results—COVID-19 should lead us to accord more value to the lived experiences of health and illness and how such experiences are differentiated across bodies, time and space. We have a unique opportunity to integrate into medicine some of the perspectives developed within the medical humanities, in particular additional epistemologies including experiential knowledge. Here, we demonstrate how the medical humanities can help navigate this nexus of science, politics and publics through two examples: experiencing disease and experiencing knowledge.^1^

## Experiencing disease

The COVID-19 pandemic demanded action, both medical and political, without a solid grounding that meeting the epistemic standards of evidence-based medicine. The urgent ‘scientific response’ to build evidence rapidly and responsively, was a process that enabled a re-assessment of experiential knowledge within medical science. The medical case history, relegated to the bottom of the evidence hierarchy since the rise of the randomised control trial and meta-analyses, witnessed a revived value as clinicians faced a new disease and very limited data. A significant outcome of this was the quick recognition that people with low-level symptoms experienced disruptions to taste and smell. This observation was mediated by a mix of social media users and medical professionals reporting and sharing case histories across a number of countries (Marinosci et al., 2020). Medical science was agile in mounting large studies so that, within weeks, the experiential knowledge was complemented with statistical evidence and anosmia was recognized as one of three key symptoms of the disease.

Similarly, online communities for sufferers of ‘long-COVID’ prompted attention to and named the condition (Callard & Perego, 2021). While similar calls from sufferers of viral related fatigue conditions pre-COVID often met neglect and trivialisation (de Wolfe, 2009), ‘long-COVID’ was quickly recognised by the medical community and the diagnosis adopted by the WHO. The scarcity of evidence regarding the new virus likely contributed to this unusual openness, but other social and contextual factors also played a role. Patient voices are often dismissed when a disease disproportionately affects disadvantaged communities, but in the case of COVID-19, health professionals were an important high status, high risk and badly affected group. Doctors, nurses and other health workers documented their experiences across various media, including leading medical journals (Garner, 2020). Their double roles as patients and professionals and the highligthing of their experential knowledge in these forums effectively bridged the spheres of science, politics and publics.

## Experiencing knowledge

Despite the meagre COVID-19 evidence base, the ‘political response’ repeatedly justified radical control measures on the grounds of ‘following the science’ (Stevens, 2020). However, scientific knowledge cannot stand in for ethical valuation and political vision; a narrow focus on bioscientific statistical abstraction with little input from the humanities and social sciences omits insights crucial for effective epidemic management (Vermeir, 2020). Here, the histories of past epidemics are instructive in documenting recurrent tensions between science, politics and publics in which relations of inequality, power and trust are central to shaping different versions of meaning around a disease. Population groups subjected to repeated historical and ongoing discrimination leading to entrenched disadvantage may well treat communications from those in power with suspicion, imputing malign interests and intentions and potentially, finding expression through conspiracy theories, conflict and sometimes violence (Snowden, 2019).

These meaning-making processes, observed in previous epidemics, have played out with COVID-19. Invoking ‘the science’ and masking the political nature of pandemic response may well contribute to further polarizing trust in both science and governance along socially differentiated lines. Despite an overall increase of trust in governments, the COVID-19 pandemic generated its own conspiracy claims of pandemic hoax, intentional infection and political interests served by protection measures, expressed through protest and even violence. For those living with existential anxiety, social alienation and powerlessness, rejecting official accounts and structures may help regain a sense of control and resilience in the face of marginalisation and exclusion (Douglas et al., 2019). A sense of insecurity is aggravated by governance strategies that target the behaviours and places of precarity (Johnson-Schlee, 2019). Government responses that implore citizens to ‘follow the science’, without accounting for the socio-culturally structured experiences of the pandemic, risk exacerbating a socio-epistemic disjuncture in which conspiracies flourish.

Our examples of experiencing disease and experiencing knowledge prompt reflection on the place of experiential knowledge in the nexus of science, politics and publics.The first reveals how first hand accounts of what it means to live with ill-health have a crucial place in medical knowledge generation. It also, however, draws attention to the social and cultural forces that inform whose voices are legitimated in which places and at what times. The abilty of patient voices to be heard in relation to COVID-19 reflected a particular bridging of science, politics and publics that is not commonly seen. The second reveals the importance of how structural and historical inequalities inform trust which in turn is central in meaning making and knowing disease. The privileging of one type of knowledge, ‘the science’, over all other ways of knowing erodes trust by ignoring differentiated experiences of disease and knowledge across divides of history, inequality and discrimination. There are, however, uncomfortable parallels of social embeddedness to confront between experiential knowledge and conspiracy theories. Conspiracy theories, like asserting the value of experiential knowledge, pose a challenge to dominant forms of knowledge. Both phenomena are supported through the sharing potential of social media and demonstrate the limitations of a simplified ‘follow the science’ creed.

This provocation, then, argues that COVID-19 has helped see more clearly a recurrent socio-epistemic crisis that requires greater recognition of three ways we build knowledge. First, the multiplicity in meaning making and knowledge formation about disease. Second, the centrality of experiential concerns of trust and discrimination, voice and public participation in how we negotiate the interactions of science, politics and publics. Third, the need to engage experience beyond the individual and to see it as inextricably imbricated in social and cultural relations of power and inequality.

## Footnotes

1 This note belongs to the topical collection “Seeing clearly through COVID-19: current and future questions for the history and philosophy of the life sciences” edited by G. Boniolo and L. Onaga.

## References

[CR1] Callard F, Perego E (2021). How and why patients made long Covid. Social Science and Medicine.

[CR2] de Wolfe P (2009). ME: The rise and fall of a media sensation. Medical Sociology Online.

[CR3] Douglas KM, Uscinski JE, Sutton RM, Cichocka A, Nefes T, Ang CS, Deravi F (2019). Understanding conspiracy theories. Political Psychology.

[CR4] Garner, P. (2020). Don’t try to dominate this virus, accommodate it. *The BMJ Opinion*. 4 Sept.https://blogs.bmj.com/bmj/2020/09/04/paul-garner-on-long-haul-covid-19-dont-try-and-dominate-this-virus-accommodate-it/ (accessed June 20, 2021)

[CR5] Johnson-Schlee S (2019). Playing cards against the state: Precarious lives, conspiracy theories, and the production of ‘irrational’ subjects. Geoforum.

[CR6] Marinosci A, Landis BN, Calmy A (2020). Possible link between anosmia and COVID-19: Sniffing out the truth. European Archives of Oto-Rhino-Laryngology.

[CR7] Snowden FM (2019). Epidemics and society: From the black death to the present.

[CR8] Stevens A (2020). Governments cannot just ‘follow the science’ on COVID-19. Nature Human Behaviour.

[CR9] Vermeir K (2020). Editorial: Doing history in the time of COVID-19. Centaurus.

